# Risk and protective factors of Leishmaniasis in the rural area of the western border region of Rio Grande do Sul, Brazil

**DOI:** 10.1186/s12917-021-03021-6

**Published:** 2021-10-14

**Authors:** Gabriela Döwich Pradella, Claudia Acosta Duarte, Taiane Acunha Escobar, Luísa Zuravski, Geórgia Camargo Góss, Jovito Adiel Skupien, Irina Lübeck

**Affiliations:** 1 Federal University of Pampa- UNIPAMPA, 118, BR 472 - Km 585, Uruguaiana, RS 97501-970 Brazil; 2 Graduate Program in Health and Life Sciences-Franciscan University – UFN, Andradas Street, 1614, Santa Maria, RS 97010-032 Brazil

**Keywords:** Canines, horses, *Leishmania* spp., PCR, Public health

## Abstract

**Background:**

The Leishmaniases are on the top of the global list of tropical neglected diseases. The number of infected dogs in South America is estimated in millions and correlated to disease cases in humans, especially in Brazil. Equines may get infected too and can play a role in the epidemiological chain. Thus, the aim of the present study was to evaluate risk and protective factors of leishmaniasis in rural areas of the western border region of Rio Grande do Sul state, Brazil by *Leishmania* spp. protozoa molecular detection and serological evaluation (ELISA) in equine and canine blood samples. This work included nine farms around the city of Uruguaiana. Epidemiologic information regarding farm characteristics and biologic material collection of canine (22) and equine (91), totalizing 113 samples was collected. The polymerase chain reaction (PCR) technique was used to detect *Leishmania* spp. in biological samples. Variables related to the farm were collected and evaluated through descriptive analysis followed by chi-square test and a logistic regression analysis.

**Results:**

Nineteen positive samples (19/113 – 16,81%) were detected, being 18 equines and 1 canine, in six of the nine farms included in the study. No animal showed clinical signs of the disease. According to the variables analyzed, when compared each characteristic separately, the presence of abundant vegetation and poor hygiene demonstrated to be risk factors to *Leishmania* infection in rural areas. The logistic regression showed that excellent general hygiene, proximity to the weir and trimmed grass were protective factors (p=0.038, p=0.001 and p=0.014, respectively). Having excellent hygiene represents a 70% lower chance of getting infected, keeping the grass cut protects the animal by more than 90% and the proximity of the weir represents a protective factor of 96%.

**Conclusions:**

The presence of *Leishmania* infection in the western border region of Rio Grande do Sul was 16,81% and it was influenced by farm characteristics. The role of the excellent general hygiene as a protective factor is extremely relevant in the leishmaniases prevention.

**Supplementary Information:**

The online version contains supplementary material available at 10.1186/s12917-021-03021-6.

## Background

Visceral leishmaniasis (VL) is caused by intracellular protozoa of the genus *Leishmania* [1]. In Brazil, the *Leishmania infantum* (synonymous with *L. chagasi*) causes the diseases and, the main species of sand flies involved in the transmission of VL are *Lutzomyia longipalpis* and *Lutzomyia cruzi* [2]. These are vectors that adapt easily to temperature changes and have twilight and nighttime activity [3]. Recently, it was reported the *Leishmania* infection in canines and humans in regions without the identification of *L. longipalpis*. In these cases, transmission occurred close to forest fragments and, after an entomological survey, 437 sandflies of two species, *Pintomyia fischeri* and *Migonemya migonei,* were infected by *L. infantum*, possibly new leishmaniases vectors [4].

Leishmaniases are found in focal areas of more than 90 countries in the tropics, subtropics, and southern Europe, on all continents except Antarctica [1, 5–7]. In Brazil, until the end of the 1980s, it was restricted to rural areas in the northeast of the country and the transmission cycle occurred between vectors and wild animals. From 1990 onwards, there was a geographic expansion to the southern states of the country and the urbanization process of the vector in different regions [8].

Rio Grande do Sul, a Brazilian state, was VL-free area until 2008. However, that year, a dog from the city of São Borja was diagnosed with canine VL (CVL). In the same period, the first autochthonous cases of human VL (HVL) occurred in Brazilian municipalities bordering Argentina (Additional file 7) [9]. Since then, this region has been considered a transmission area, with canine and human cases and the presence of the main vector, *L. longipalpis* [10]. In the following years, the disease spread to other state regions and, in 2016, the first case in humans was recorded in the capital, Porto Alegre [4]. In Uruguaiana, since 2011, when the first human case occurred in urban areas, the parasite was also detected in canines and horses [11–13] and, recently, the disease has spread to rural areas [14].

Animal species are important for disease epidemiological cycle and maintenance of the infection. The domestic dog is considered a reservoir of VL in urban centers, however, other mammals, including rodents, primates, felines, and horses can get infected [7, 12, 15–17] and act both as possible reservoirs and as a source of blood for sand flies [18].

Among the risk factors for infection and disease, exposure to the vector can be highlighted. They can adapt easily to different biotopes [19], and the presence of other animal species, organic matter and green areas can also assist in the sand fly maintenance and perpetuation [20, 21]. In 2018, the analysis of risk factors associated with CVL in Uruguaiana identified that, in residences that contained more than 10m^2^ of green area in the home had a greater chance of exposure to leishmaniases. Although, other socioeconomic and environmental variables (presence of basic sanitation, garbage collection, green area around the home, presence of leafy trees, proximity to the Uruguay River, presence of other animal species, etc.) were analyzed, there was no correlation of these characteristics to the greater risk of acquiring the infection. It happened because the city is considered a transmission area for CVL with distribution in practically all neighborhoods, regardless of environmental and socioeconomic conditions [21].

Uruguaiana city is part of a triple border with Uruguay and Argentina, becoming important in the epidemiological view. Uruguay is considered a potential risk of VL transmission due to the following factors: presence of *L. longipalpis* in Salto and Bella Unión, border with Barra do Quaraí city/Brazil; presence of VL from Argentina and Brazil bordering regions; and movement of people and dogs across the border [22]. In Argentina, seven locations are classified as vulnerable, six in the province of Corrientes, amongst them Paso de los Libres city that is bordered with Uruguaiana city [23].

In the meantime, to study factors that may contribute to the characterization of environments that favor the occurrence of VL, as well as the identification of infection in different animal species can enable the knowledge of the dynamics of infection in each region, facilitating the adoption and improvement of more effective control and prevention measures. In this sense, the objective of the present study was to evaluate risk and protective factors of leishmaniasis in farms on the western border of Rio Grande do Sul, a triple border region between Brazil/Argentina/Uruguay.

## Results

From the 113 samples collected (91 horses and 22 canines) 19 (16,81%, 18 horses and 1 dog) were positive in PCR and/or ELISA tests. With the list of equine samples 7 out of 91 (7.69%) were positive in the PCR using RV1/RV2 primers. Sequencing analysis, performed on a horse positive sample, enabled the identification of major similarity with the species *L. infantum* (GenBank accession 103741.1; query cover 96%; ident 93%), as previously published by the group [14]. Out of 91 horses, 13 (14,28%) were positive in an in-house ELISA test, as described in the methodology. Only two horse samples were positive in both diagnostic tests (PCR and ELISA). The canine sample totaled 22 animals, of these 1 (4,54%) was positive in PCR technique. Due to the limitations of the collection, only 5 dogs out of 22 had a sufficient sample to perform ELISA and they were all negative. All positive animals (18 horses and 1 canine) to *Leishmania* spp. in the PCR and/or ELISA were healthy, with no clinical signs at the time of material collection. The positive samples belonged to six properties in the rural area, identified on the map as: 1, 2, 5, 7, 8 and 9 (Additional file 5: Fig. 1).

Table 1 demonstrates a relationship with the factors: presence of abundant vegetation (OR: 2.757; p=0.043) and the lack of hygiene in the property (OR: 5.416; p<0.0001) as potential risk factors for the occurrence of infections. It can also be observed that at the properties, which did not present any positive animal, they did not own dogs in the place, or these animals were vaccinated for leishmaniasis.

**Table 1:** List of variables evaluated in each of the properties included in the study

Exposure to characteristic	PCR and / or ELISA negative animals (n = 94)	PCR and / or ELISA positive animals (n = 19)	Odds ratio (OR)	Confidence interval (CI) (95%)	p Value
**Proximity to the weir**					
Yes	41	11	1.777	0.327	0.254
No	53	8			
**Proximity to River**					
Yes	26	3	0.490	0.090	0.279
No	68	16			
**Proximity to dam**					
Yes	79	9	0.170	0.031	0.000
No	15	10			
**Proximity to lakes / streams**					
Yes	26	0	0	-	0.008
No	68	19			
**Presence of organic matter**					
Yes	59	8	0.431	0.079	0.094
No	35	11			
**Presence of abundant vegetation**					
Yes	27	10	2.757	0.508	0.043*
No	67	9			
**Grass**					
Yes	85	15	0.397	0.073	0.152
No	9	4			
**Shading in the field**					
Yes	10	1	0.466	0.086	0.470
No	84	18			
**Eucalyptus forest**					
Yes	36	4	0.429	0.079	0.151
No	58	15			
**Open containers with water**					
Yes	49	8	0.667	0.123	0.425
No	45	11			
**Poor hygiene on the property**					
Yes	16	10	5.416	0.998	0.000*
No	78	9			
**Trees in the yard**					
Yes	51	14	2.360	0.435	0.118
No	43	5			
**Proximity to forests**					
Yes	55	14	1.985	0.366	0.216
No	39	5			
**Inappropriate waste destination**					
Yes	73	18	5.178	0.954	0.086
No	21	1			
**Vaccination for leishmaniasis**					
Yes	16	0	0	-	0.052
No	78	19			
**Insecticide collar**					
Yes	0	0	-	-	-
No	94	19			
**Use of antiparasitic**					
Yes	94	19	-	-	-
No	0	0			
**Use of insecticides**					
Yes	69	18	6.521	1.202	0.043*
No	25	1			

*Variables with significant statistical difference when compared positive and negative samples, OD ≥ 1 and p≤ 0.05

(-) calculation did not work because it divides the value by zero, it would need a larger sample to be able to include these variables

Due to the large number of variables collected, regression models were created and those that presented statistically significant values in more than one model were used in the final regression model. Some variables, not conditioned to the *Leishmania* infection, were excluded from the analysis. Thus, excellent general hygiene, proximity to the weir and trimmed grass were protective factors (p=0.038, p=0.001 and p=0.014, respectively). Having excellent hygiene represents a 70% lower chance of the animal getting sick, keeping the grass cut protects the animal by more than 90% and the proximity of the weir represents a protective factor of 96% (Table 2).

**Table 2:** Regression models for the variables that presented statistically significant values in more than one model used in the final regression

Variable	Odds Ratio	95% Confidence Interval		p Value
		Lower	Upper	
**Garbage destination**	0.607	0.061	6.014	0.670
**General hygiene**	0.293	0.092	0.935	0.038
**Proximity to Weir**	0.040	0.006	0.284	0.001
**Trimmed grass**	0.099	0.016	0.623	0.014

## Discussion

The observed frequency of infected animals in the rural area was 19,78% for horses and 4,54% for dogs. In Uruguaiana, it was observed a 39,06% (75 of 192 samples) of infected horses in urban area from 10 neighborhoods using molecular biology technique [13]. In relation to dogs from urban areas, 57.48% (261 of 454) were confirmed positive by the team of the LACEN – RS (Rio Grande do Sul – RS, Central Laboratory) through serological tests [21]. It was possible to note that, in rural areas of the city the frequency of positive animals is still lower than urban areas. This study started the rural area investigation and made it possible to define the importance of this studied area.

Despite the urbanization of leishmaniases, the preservation of rural characteristics still exists [8]. Studies show that animal husbandry can influence local health conditions due to the production of organic waste, which favors the attraction and maintenance of the vector in the environment [20]. Some results observed such as tall grass, presence of green area, creation of other animals and presence of organic matter can influence its perpetuation [21, 24]. Until 2008, the south region of Brazil was free from leishmaniasis. After that, VL in human and dog occurred and the presence of the phlebotomine vector was identified [25]. In 2017, 32 CVL in Barra do Quaraí city and 25 CVL in Itaqui city were registered at LACEN/RS, both neighboring of Uruguaiana, with 99 CVL cases [26].

The role of dogs in leishmaniases biological cycle is well elucidated. In Uruguaiana, leishmaniasis in dogs can be considered endemic because it is present in all neighborhoods of the city [21]. On the other hand, infection in horses has been shown to be relevant in several regions of Brazil and the understanding regarding participation in the cycle and the possibility of maintaining the disease in the environment is still an obstacle. A study demonstrated a seroprevalence of 48.0% (73/152) of *Leishmania* spp*.* in horses from south Brazil sent to two slaughterhouses registered with the Federal Inspection Service [27]. The investigation of seroprevalence in Araçatuba (14,59% [68/466]) and Uberlândia (24.1% [62/257]), two Brazilian cities located in the center of the country, also demonstrated positive results and the need of further investigation to determine the real importance of horses in the infection cycle [28, 29].

In Uruguaiana urban perimeter, the presence of green areas was associated with higher CVL occurrence [21], like the findings of the present study. This data could be supported by the vector characteristic analyzed by entomologist study where the immature phlebotomine sand flies are present in soil of tropical forests [21]. The definition of poor hygiene as a risk factor and adequate hygiene as a protective factor was grateful when control alternatives were studied. Similarly, elucidation of trimmed grass and proximity with weir as protective factor can help in control strategies. The one health leishmaniases control was evaluated in a study and, the multiple approach, without culled dogs, was showed as an important prevention method [30, 31]. Epidemiological studies became necessary to define, in each local, the relevant factors that influence in the maintenance of the vector.

The study limitations were related to the agreement of owners to participate and to allow the sample collection. Although the number of animals collected was not so great, it allowed the observation of animals from farms with main access through the three main access roads to the municipality and the border with Argentina and Uruguay. Another limitation of the study is related to economic and personnel difficulties for a broader approach due to the distance and difficulty accessing places. However, it allows to visualize the presence of asymptomatic *Leishmania* infection in rural areas of Brazil western border and to start the epidemiological analysis related to the disease in this location of the country.

## Conclusions

It was possible to conclude the importance of evaluating epidemiological variables to determine the risk and protective factors related to the maintenance of the asymptomatic *Leishmania* infection in rural areas, as well as the importance of equine species in leishmaniasis epidemiology. The general hygiene and trimmed grass, showed as protective factors, enable the intervention in leishmaniasis disease with a multi varied prevention.

## Methods

The protocol was performed in accordance with the relevant guidelines and regulations. The work was carried out from July to December 2017 at rural areas of Uruguaiana city. Located in the extreme west of Rio Grande do Sul, Brazil, at 643 km from Porto Alegre, the capital of the State. The city is bathed by the Uruguay River, which forms a border with Argentina (Paso de los Libres city/province of Corrientes) and Uruguay far away 80Km (Bella Union/department of Artigas). The municipality has 125.435 inhabitants, 117.415 living in urban areas and 8.020 in rural areas. It has 5.702.098 km^2^ total area and has as main aptitude the extensive breeding of cattle, horses and sheep and as agricultural production, the rice monoculture [32].

Randomly, nine properties located in Uruguaiana, in the southwest (Brazil/Uruguay/Argentina border), northeast (Brazil/Argentina) and east (Brazil, main road to the center of the state), were selected and identified by numbers (Additional file 5: Fig. 1). The properties included in the study were contacted through owners registered at the University Hospital of Unipampa and after the acceptance of the animal guardians. The coordinates were not exposed to maintain the identity of the properties and the confidentiality of the information obtained. Access to the study sites was basically composed of a main paved road (BR 290 and BR 472) and secondary land accesses.

Epidemiological variables, information about the location, such as the size area of the property, the number of employees and its suitability (main purpose) and environmental variables (Additional file 6: Fig. 2), as proximity or presence of river, weir or dam, presence of vegetation around the headquarters, presence of water tank and/or containers without a lid, presence of organic matter, destination of garbage, presence of other animal species, vaccination and use of insecticidal collar on dogs, among others, were observed and registered in each property visited.

The properties included in the study had an average of 1200 hectares (ha) (minimum 54 and maximum 3656) of which: three smaller than 100ha, two between 100 and 1000ha and four properties larger than 1000ha. The main activity of the locals was, in their majority, beef cattle, followed by horse breeding and dairy cattle (Table 3).

**Table 3:** Information about farm characteristics and collected animal numbers from each farm

Farm number	Size (acre)	Main road reference	Main activity	n horse	n canine
1	3656	BR 472 to Barra	Beef cattle	10	2
2	363	BR 472 to Barra	Beef cattle and sheep	10	3
3	1900	BR 472 to Barra	Beef cattle	10	0
4	2001	BR 290 to Alegrete	Beef cattle	10	0
5	54	BR 290 to Alegrete	Dairy cattle and swine	10	3
6	1312	BR 290 to Alegrete	Beef cattle	11	5
7	609	BR 472 to Itaqui	Beef cattle	7	4
8	87	BR 472 to Itaqui	Horse breeding	12	3
9	3045	BR 472 to Itaqui	Beef cattle and Rice	11	2
		**Total**		**91**	**22**

113 animals were analyzed, including 91 horses and 22 dogs. The equine sample size was calculated based on expected prevalence, confidence level and error. The expected prevalence for rural areas was previously reported and, for equine species, it was 6.19% [14]. The applied formula included sample size, Z statistic (1.96 for 95% confidence level), expected proportion (6.19%) and precision/error (5%) [33]. The calculated sample size was 89 equines and 91 were included in this study. The dog sample number was determined by the availability of animals in the properties, as an addition to the work, because of the lack of data referring to the number of animals in rural areas. Each animal received a number, and a clinical examination and identification was carried out by filling in data on clinical files for horses and canines. The clinical examination included: heart rate, respiratory rate, mucous membranes, rectal temperature, and lymph node palpation, in addition to abdominal palpation in dogs. The presence of alopecia, injuries and weight loss were also observed. Blood was collected by external jugular venipuncture in horses and external jugular or cephalic in canines. The blood samples were collected in two different tubes, EDTA (containing anticoagulant) and serum separator tube (SSP) and placed in thermal boxes with ice to transport to the Laboratory of Veterinary Microbiology at UNIPAMPA- Uruguaiana/RS, where they were processed.

DNA extraction was carried out according to the Salting-Out technique [34] and amplification of genetic material to determine positive samples for *Leishmania* spp. The PCR amplification was carried out with the primers RV1 (5’-CTTTTCTGGTCCCGCGGGTAGG-3’) and RV2 (3’-CCACCTGGGCTATTTTACACCA–5’), target sequence of the LT1 fragment, with 145 bp to identify *Leishmania* spp. belonging to the donovani complex [35]. PCR conditions were described elsewhere [36, 37]. The reaction mixtures adjusted in a final volume of 25 μl (25-50 ng of DNA), 2.5 U of Taq DNA polymerase, 1X PCR buffer, 1.5 mM of MgCl2, (Invitrogen, USA), 10 mM of each dNTP (dATP, dCTP, dGTP, and dTTP) (Promega, EUA),10 pmol of each primer (IDT, EUA) and ultrapure water, were performed in thermocycler (Amplitherm Thermal Cyclers). All assays included negative PCR controls (PCR mix without DNA), and positive PCR controls (DNA extracted from *L. (L.) infantum* promastigotes (MHOM/BR/1974/PP75). The results were visualized by electrophoresis in 1.5% agarose gels in 1 × TAE-buffer staining with ethidium bromide [2].

RV1/RV2 PCR product from one horse positive sample was confirmed by direct sequencing, performed by an automated sequencer ABI-Prism 3500 Genetic Analyser (Applied Biosystems, USA), after purification with Purelink™ Quick Gel Extraction and PCR Purification kit (Invitrogen, USA) [11].

The ELISA protocol was performed, according to protocols already described, with modifications [33, 38], using *L. infantum* grown in the laboratory to the fixation. First, the plates were pre-coated with 10μg/mL *L. infantum* antigen. Initially, the 1st antibody dilution (serum of the animal to be tested) was diluted in phosphate-saline dilution buffer (PBS) supplemented with 0.05% polysorbate 20 (PBS Tween) and 0.25% casein, at a concentration 1/200 and a volume of 100 μl /well. The plate was gently shaken and incubated for 45 minutes at 37°C (Thermo-Shaker Agimaxx®). After that, a wash in a microplate washer (Wellwash- Thermo Fisher Scientific®) with PBS tween was performed using four cycles. The next step was the addition of 100 μl /well of the 2nd antibody, specie specific (Goat Anti-Dog IgG H&L [HRP] ab112852 and Rabbit Anti-Horse IgG H&L [HRP] ab102405- Abcam®) in PBS Tween. The concentration used was 1/10000. The plate was gently stirred and incubated for 45 minutes at 37°C, again. The last wash was performed in a microplate washer with PBS tween using four cycles, and 100 μl/well of the substrate solution (Tetramethylbenzidine- TMB) was added and incubated for 15 minutes in the dark. The reaction was finalized with the addition of 25 μl /well of the stop solution (Sulfuric Acid 2M). Finally, the reading was performed according to the manufacturer's instructions of the development solution (TMB) with an ELISA reader (Multiskan FC- Thermo Fisher Scientific®) at 450nm.

The ideal conditions for the immunodiagnostic assay's performance were obtained from the mean absorbance of positive and negative controls (signal-to-noise). The cut-off was determined with the mean absorbance of negative controls plus three times the standard deviation of negative controls [38]. It was established negative and positive samples if the absorbance value was 10% below or above the cut-off. Samples with values inside the cut-off determination were considered undetermined. The signal to noise was 3.496; cut off 0.360; negative control means 0.246 and positive control means 0.860.

The group of animals, dogs and horses, was considered positive by the detection, via molecular biology, of genetic material from *Leishmania* sp. and by enzyme-linked immunosorbent assay (ELISA) serological method (protocol in house). Positive animals were those that presented a positive test regardless of the technique used.

A statistical analysis was performed using SPSS for MAC software, version 24 (SPSS Inc, Chicago, IL, USA). Initially, a descriptive analysis of the animals was performed, followed by the chi-square test. The strength of association between the occurrence of infection and the presence of variables was calculated by odds ratio, using a 95% confidence interval. In addition, a logistic regression analysis was applied, considering a significance level of 5%. The final model was used after several models were created, where only the variables statistically significant remained.

## Supplementary Information

**Additional file 1.**

**Additional file 2.**

**Additional file 3.**

**Additional file 4.**

**Additional file 5. Fig. 1.** Demonstrative map of the distribution of rural properties on the Western Frontier of Rio Grande do Sul (numbered from 1 to 9) where data and biological material collections were carried out. Maps data: Google Earth, © 2020 CNES/Airbus, Maxar Technologies.

**Additional file 6. Fig. 2.** Epidemiological form used to register the environmental variables.

**Additional file 7.**

## Data Availability

The data supporting the conclusions of this article are included within the article. The PCR and ELISA data are registered at laboratory book and could be solicited to the authors. The epidemiological forms and individual clinical forms from canine and equine are scanned and stored online.

## Abbreviations

CVL: Canine visceral leishmaniasis
EDTA: Ethylenediaminetetraacetic acid
ELISA: Enzyme-linked immunosorbent assay
HVL: Human visceral leishmaniasis
LACEN: State public health laboratory
PBS: Phosphate-saline dilution buffer
PCR: Polymerase chain reaction
SPSS: Statistical package software
SSP: Serum separator tube
TMB: Tetramethylbenzidine
VL: Visceral leishmaniasis
