# Supplementary material for: Risk and protective factors of Leishmaniasis in the rural area of the western border region of Rio Grande do Sul, Brazil
Source: BMC Vet Res. 2021 Oct 14;17:330. doi: 10.1186/s12917-021-03021-6 (PMC8515718; doi:10.1186/s12917-021-03021-6)
Supplement: Supplementary file 1 — Additional file 1. [file 12917_2021_3021_MOESM1_ESM.pdf]

MINISTÉRIO DA EDUCAÇÃO
FUNDAÇÃO UNIVERSIDADE FEDERAL DO PAMPA
(Lei nº 11.640, de 11 de janeiro de 2008)

Pró-Reitoria de Pesquisa, Pós-Graduação e Inovação (PROPII)

COMISSÃO DE ÉTICA NO USO DE ANIMAIS - CEUA

Fone: (55)3911-0200, E-mail: ceua@unipampa.edu.br

CERTIFICADO DE APROVAÇÃO DE PROTOCOLO PARA USO DE ANIMAIS EM PESQUISA

Número de protocolo da CEUA: 022/2017

Título: Identificação e caracterização molecular de *Leishmania sp.* em animais domésticos.

Data da aprovação: 03/07/2017

Período de vigência do projeto: 03/07/2019

Pesquisadores(a): Irina Lubeck

Campus: Uruguaiana

Telefone: (55) 99948-7512

E-mail: irinalubeck@unipampa.edu.br

CEUA
Comissão de Ética em Uso de Animais

**Finalidade**	**( ) Ensino (X) Pesquisa**
**Espécie/Linhagem/Raça**	Caninos, Felinos e Equinos
**Nº de animais**	135, 135 e 192
**Peso/Idade**	10, 5 e 320 Kg / 2 a 15 anos
**Sexo**	Machos e Fêmeas
**Origem**	Bairros da zona urbana e rural dos municípios de Uruguaiana e Barra do Quaraí

Profª. Drª. Vanusa Manfredini
Coordenadora CEUA/UNIPAMPA
