# Supplementary material for: Risk and protective factors of Leishmaniasis in the rural area of the western border region of Rio Grande do Sul, Brazil
Source: BMC Vet Res. 2021 Oct 14;17:330. doi: 10.1186/s12917-021-03021-6 (PMC8515718; doi:10.1186/s12917-021-03021-6)
Supplement: Supplementary file 2 — Additional file 2. [file 12917_2021_3021_MOESM2_ESM.pdf]

Pradella, Gabriela Döwich
Federal University of Pampa
Uruguaiana- Brazil

January, 19

Dear reviewer,

This declaration confirm that our protocol was performed in accordance with the relevant guidelines and regulations and was approved by an appropriate ethics committee. The research was approval by the Animal Use Ethics Commission (CEUA) of the Federal University of Pampa (UNIPAMPA) under protocol number 031/2017. The farms included were in accordance with consent of owners.

gabrieladowich@hotmail.com

Thank you.

Sincerely,

Gabriela Döwich Pradella
