# Supplementary material for: Risk and protective factors of Leishmaniasis in the rural area of the western border region of Rio Grande do Sul, Brazil
Source: BMC Vet Res. 2021 Oct 14;17:330. doi: 10.1186/s12917-021-03021-6 (PMC8515718; doi:10.1186/s12917-021-03021-6)
Supplement: Supplementary file 3 — Additional file 3. [file 12917_2021_3021_MOESM3_ESM.pdf]

MINISTÉRIO DA EDUCAÇÃO
FUNDAÇÃO UNIVERSIDADE FEDERAL DO PAMPA
(Lei nº 11.640, de 11 de janeiro de 2008)

CEUA
Comissão de Ética em Uso de Animais

Pró-Reitoria de Pesquisa, Pós-graduação e Inovação

COMISSÃO DE ÉTICA NO USO DE ANIMAIS - CEUA
Fone: (55) 3911-0200, E-mail:
ceua@unipampa.edu.br

TERMO DE CONSENTIMENTO
(conforme Resolução Normativa do CONCEA nº 22/2015)

Título do projeto:

Identificação e caracterização de *Leishmania* sp. em animais domésticos

Nome do pesquisador principal:

Irina Lubeck

Razão social e CIAEP instituição da CEUA que aprovou:

Universidade Federal do Pampa

Objetivos do estudo:

Geral

Verificar a ocorrência de *Leishmania* sp., em caninos, equinos e felinos de zona urbana e rural dos municípios de Barra do Quaraí e Uruguaiana – RS.

Específicos:

Verificar a presença de material genético de *Leishmania* sp. em amostras de sangue periférico e lesões de pele dos caninos, equinos e felinos, através da técnica de PCR;
Verificar a presença de material genético de *Leishmania* sp. em amostras de sangue periférico e lesões de pele dos caninos, equinos e felinos, através da técnica de qPCR;
Realizar a caracterização molecular em amostras positivas para verificar as espécies encontradas;
Avaliar os aspectos clínicos dos animais naturalmente infectados, visando um maior conhecimento acerca das possíveis manifestações clínicas;
Investigar a presença de anticorpos anti-*Leishmania* sp. nos caninos, equinos e felinos;
Investigar a presença de anticorpos anti-*Leishmania* sp. via ensaio de triagem imunocromatográfico Teste Rápido Dual Path Platform nos caninos;
Comparar as técnicas sorológicas e moleculares nas espécies animais estudadas.

Procedimentos a serem realizados com os animais:

Serão coletadas amostras de sangue via venopunção e coleta de suabes oculares para determinação da infecção por leishmania por métodos moleculares e sorológicos como também, será realizado exame clínico que consta de: aferição da frequência cardíaca, frequência respiratória, tempo de perfusão capilar, grau de hidratação, motilidade intestinal, inspeção de linfonodos, pulso digital e temperatura retal. Para tanto, será realizada somente uma coleta/visita. Animais que apresentem alterações clínicas como infartamento de linfonodos, se efetuada punção para realização de citologia. Em caso da ocorrência de lesões de pele, os animais serão encaminhados para o Hospital Veterinário Universitário da Unipampa (HUVet) para a realização de biópsia de pele.

Potenciais riscos para os animais:

Não são apresentados riscos aos animais

Cronograma:

Atividade	Set	Out	Nov	Dez	Jan	Fev	Mar	Abr	Mai
Coleta de felinos e caninos	X	X	X	X	X	X	X	X	X

Benefícios:

Os animais serão submetidos a exame clínico completo e avaliação por médico veterinário capacitado, permitindo a detecção de alterações clínicas e encaminhamento para investigações complementares. Adicionalmente, os animais são submetidos a testes para detecção de *Leishmania* ou resposta à mesma, fornecendo ao tutor informação sobre a situação de seu animal em relação a leishmaniose visceral e propiciando ao mesmo a tomada de ações com base no resultado do diagnóstico. As informações obtidas alertarão a população sobre a adoção de medidas preventivas, manejo com animais domésticos e manejo ambiental nas residências, evitando a disseminação do vetor e a ocorrência da enfermidade.

Esclarecimentos ao proprietário sobre a participação do animal neste projeto:

- Sua autorização para a inclusão do (s) seu (s) animal (is) nesse estudo é voluntária.
- Seu (s) animal (is) poderá(ão) ser retirado (s) do estudo, a qualquer momento, sem que isso cause qualquer prejuízo a ele (s).
- A confidencialidade dos seus dados pessoais será preservada.
- Os membros da CEUA ou as autoridades regulatórias poderão solicitar suas informações, e nesse caso, elas serão dirigidas especificamente para fins de inspeções regulares.

O Médico Veterinário responsável pelo (s) seu (s) anima (is) será o (a) Dr (a) Irina Lübeck, inscrita (a) no CRMV sob o n 8926. Além dele, a equipe do Pesquisador Principal Claudia Acosta Duarte também se responsabilizará pelo bem estar do (s) seu (s) animal (is) durante todo o estudo e ao final dele.
Quando for necessário, durante ou após o período do estudo, você poderá entrar em contato com o Pesquisador Principal ou com a sua equipe pelos contatos:

Tel. de emergência: (55) 999487512

Declaração de consentimento

Fui devidamente esclarecido (a) sobre todos os procedimentos deste estudo, seus riscos e benefícios ao (s) animal (is) pelo (s) qual (is) sou responsável. Fui também informado que posso retirar meu (s) animal (is) do estudo a qualquer momento. Ao assinar este Termo de Consentimento, declaro que autorizo a participação do (s) meu (s) animal (is) identificado (s), a seguir, neste projeto.

Local e data: Uruguaiana, Julho 2017.

Assinatura do Pesquisador: [signature]

Assinatura do Responsável: Nádia González Ribas

MINISTÉRIO DA EDUCAÇÃO
FUNDAÇÃO UNIVERSIDADE FEDERAL DO PAMPA
(Lei nº 11.640, de 11 de janeiro de 2008)

CEUA

Comissão de Ética em Uso de Animais

Pró-Reitoria de Pesquisa, Pós-graduação e Inovação

COMISSÃO DE ÉTICA NO USO DE ANIMAIS - CEUA

Fone: (55) 3911-0200, E-mail:
~~ceua@unipampa.edu.br~~

TERMO DE CONSENTIMENTO

(conforme Resolução Normativa do CONCEA nº 22/2015)

Título do projeto:

Identificação e caracterização de *Leishmania* sp. em animais domésticos

Nome do pesquisador principal:

Irina Lubeck

Razão social e CIAEP instituição da CEUA que aprovou:

Universidade Federal do Pampa

Objetivos do estudo:

Geral

Verificar a ocorrência de *Leishmania* sp., em caninos, equinos e felinos de zona urbana e rural dos municípios de Barra do Quaraí e Uruguaiana – RS.

Específicos:

Verificar a presença de material genético de *Leishmania* sp. em amostras de sangue periférico e lesões de pele dos caninos, equinos e felinos, através da técnica de PCR;
Verificar a presença de material genético de *Leishmania* sp. em amostras de sangue periférico e lesões de pele dos caninos, equinos e felinos, através da técnica de qPCR;
Realizar a caracterização molecular em amostras positivas para verificar as espécies encontradas;
Avaliar os aspectos clínicos dos animais naturalmente infectados, visando um maior conhecimento acerca das possíveis manifestações clínicas;
Investigar a presença de anticorpos anti-*Leishmania* sp. nos caninos, equinos e felinos;
Investigar a presença de anticorpos anti-*Leishmania* sp. via ensaio de triagem imunocromatográfico Teste Rápido Dual Path Platform nos caninos;
Comparar as técnicas sorológicas e moleculares nas espécies animais estudadas.

Procedimentos a serem realizados com os animais:

Serão coletadas amostras de sangue via venopunção e coleta de suabes oculares para determinação da infecção por leishmania por métodos moleculares e sorológicos como também, será realizado exame clínico que consta de: aferição da frequência cardíaca, frequência respiratória, tempo de perfusão capilar, grau de hidratação, motilidade intestinal, inspeção de linfonodos, pulso digital e temperatura retal. Para tanto, será realizada somente uma coleta/visita. Animais que apresentem alterações clínicas como infartamento de linfonodos, se efetuada punção para realização de citologia. Em caso da ocorrência de lesões de pele, os animais serão encaminhados para o Hospital Veterinário Universitário da Unipampa (HUVet) para a realização de biópsia de pele.

Potenciais riscos para os animais:

Não são apresentados riscos aos animais

Cronograma:

Atividade	Set	Out	Nov	Dez	Jan	Fev	Mar	Abr	Mai
Coleta de felinos e caninos	X	X	X	X	X	X	X	X	X

Benefícios:

Os animais serão submetidos a exame clínico completo e avaliação por médico veterinário capacitado, permitindo a detecção de alterações clínicas e encaminhamento para investigações complementares. Adicionalmente, os animais são submetidos a testes para detecção de *Leishmania* ou resposta à mesma, fornecendo ao tutor informação sobre a situação de seu animal em relação a leishmaniose visceral e propiciando ao mesmo a tomada de ações com base no resultado do diagnóstico. As informações obtidas alertarão a população sobre a adoção de medidas preventivas, manejo com animais domésticos e manejo ambiental nas residências, evitando a disseminação do vetor e a ocorrência da enfermidade.

Esclarecimentos ao proprietário sobre a participação do animal neste projeto:

- Sua autorização para a inclusão do (s) seu (s) animal (is) nesse estudo é voluntária.
- Seu (s) animal (is) poderá(ão) ser retirado (s) do estudo, a qualquer momento, sem que isso cause qualquer prejuízo a ele (s).
- A confidencialidade dos seus dados pessoais será preservada.
- Os membros da CEUA ou as autoridades regulatórias poderão solicitar suas informações, e nesse caso, elas serão dirigidas especificamente para fins de inspeções regulares.

O Médico Veterinário responsável pelo (s) seu (s) anima (is) será o (a) Dr (a) Irina Lübeck, inscrita (a) no CRMV sob o n 8926. Além dele, a equipe do Pesquisador Principal Claudia Acosta Duarte também se responsabilizará pelo bem estar do (s) seu (s) animal (is) durante todo o estudo e ao final dele.

Quando for necessário, durante ou após o período do estudo, você poderá entrar em contato com o Pesquisador Principal ou com a sua equipe pelos contatos:

Tel. de emergência: (55) 999487512

Declaração de consentimento

Fui devidamente esclarecido (a) sobre todos os procedimentos deste estudo, seus riscos e benefícios ao (s) animal (is) pelo (s) qual (is) sou responsável. Fui também informado que posso retirar meu (s) animal (is) do estudo a qualquer momento. Ao assinar este Termo de Consentimento, declaro que autorizo a participação do (s) meu (s) animal (is) identificado (s), a seguir, neste projeto.

Local e data: Uruguaiana, Dezembro de 2017

Assinatura do Pesquisador: [signature]

Thiago Cardoso
Méd. Veterinário
CRMV-RS 15183

Assinatura do Responsável: [signature]

MINISTÉRIO DA EDUCAÇÃO
FUNDAÇÃO UNIVERSIDADE FEDERAL DO PAMPA
(Lei nº 11.640, de 11 de janeiro de 2008)

Pró-Reitoria de Pesquisa, Pós-graduação e Inovação

COMISSÃO DE ÉTICA NO USO DE ANIMAIS - CEUA
Fone: (55) 3911-0200, E-mail:
ceua@unipampa.edu.br

TERMO DE CONSENTIMENTO

(conforme Resolução Normativa do CONCEA nº 22/2015)

Título do projeto:

Identificação e caracterização de *Leishmania* sp. em animais domésticos

Nome do pesquisador principal:

Irina Lubeck

Razão social e CIAEP instituição da CEUA que aprovou:

Universidade Federal do Pampa

Objetivos do estudo:

Geral

Verificar a ocorrência de *Leishmania* sp., em caninos, equinos e felinos de zona urbana e rural dos municípios de Barra do Quaraí e Uruguaiana – RS.

Específicos:

Verificar a presença de material genético de *Leishmania* sp. em amostras de sangue periférico e lesões de pele dos caninos, equinos e felinos, através da técnica de PCR;
Verificar a presença de material genético de *Leishmania* sp. em amostras de sangue periférico e lesões de pele dos caninos, equinos e felinos, através da técnica de qPCR;
Realizar a caracterização molecular em amostras positivas para verificar as espécies encontradas;
Avaliar os aspectos clínicos dos animais naturalmente infectados, visando um maior conhecimento acerca das possíveis manifestações clínicas;
Investigar a presença de anticorpos anti-*Leishmania* sp. nos caninos, equinos e felinos;
Investigar a presença de anticorpos anti-*Leishmania* sp. via ensaio de triagem imunocromatográfico Teste Rápido Dual Path Platform nos caninos;
Comparar as técnicas sorológicas e moleculares nas espécies animais estudadas.

Procedimentos a serem realizados com os animais:

Serão coletadas amostras de sangue via venopunção e coleta de suabes oculares para determinação da infecção por leishmania por métodos moleculares e sorológicos como também, será realizado exame clínico que consta de: aferição da frequência cardíaca, frequência respiratória, tempo de perfusão capilar, grau de hidratação, motilidade intestinal, inspeção de linfonodos, pulso digital e temperatura retal. Para tanto, será realizada somente uma coleta/visita. Animais que apresentem alterações clínicas como infartamento de linfonodos, se efetuada punção para realização de citologia. Em caso da ocorrência de lesões de pele, os animais serão encaminhados para o Hospital Veterinário Universitário da Unipampa (HUVet) para a realização de biópsia de pele.

Potenciais riscos para os animais:

Não são apresentados riscos aos animais

Cronograma:

Atividade	Set	Out	Nov	Dez	Jan	Fev	Mar	Abr	Mai
Coleta de felinos e caninos	X	X	X	X	X	X	X	X	X

Benefícios:

Os animais serão submetidos a exame clínico completo e avaliação por médico veterinário capacitado, permitindo a detecção de alterações clínicas e encaminhamento para investigações complementares. Adicionalmente, os animais são submetidos a testes para detecção de *Leishmania* ou resposta à mesma, fornecendo ao tutor informação sobre a situação de seu animal em relação a leishmaniose visceral e propiciando ao mesmo a tomada de ações com base no resultado do diagnóstico. As informações obtidas alertarão a população sobre a adoção de medidas preventivas, manejo com animais domésticos e manejo ambiental nas residências, evitando a disseminação do vetor e a ocorrência da enfermidade.

Esclarecimentos ao proprietário sobre a participação do animal neste projeto:

- Sua autorização para a inclusão do (s) seu (s) animal (is) nesse estudo é voluntária.
- Seu (s) animal (is) poderá(ão) ser retirado (s) do estudo, a qualquer momento, sem que isso cause qualquer prejuízo a ele (s).
- A confidencialidade dos seus dados pessoais será preservada.
- Os membros da CEUA ou as autoridades regulatórias poderão solicitar suas informações, e nesse caso, elas serão dirigidas especificamente para fins de inspeções regulares.

O Médico Veterinário responsável pelo (s) seu (s) anima (is) será o (a) Dr (a) Irina Lübeck, inscrita (a) no CRMV sob o n 8926. Além dele, a equipe do Pesquisador Principal Claudia Acosta Duarte também se responsabilizará pelo bem estar do (s) seu (s) animal (is) durante todo o estudo e ao final dele.
Quando for necessário, durante ou após o período do estudo, você poderá entrar em contato com o Pesquisador Principal ou com a sua equipe pelos contatos:

Tel. de emergência: (55) 999487512

Declaração de consentimento

Fui devidamente esclarecido (a) sobre todos os procedimentos deste estudo, seus riscos e benefícios ao (s) animal (is) pelo (s) qual (is) sou responsável. Fui também informado que posso retirar meu (s) animal (is) do estudo a qualquer momento. Ao assinar este Termo de Consentimento, declaro que autorizo a participação do (s) meu (s) animal (is) identificado (s), a seguir, neste projeto.

Local e data: Uruguaiana, julho 2017

Assinatura do Pesquisador: [signature]

Assinatura do Responsável: [signature]

Greice Schwanck Trojan
CPF 664.831.820-15
CRA/RS 012812/0

MINISTÉRIO DA EDUCAÇÃO
FUNDAÇÃO UNIVERSIDADE FEDERAL DO PAMPA
(Lei nº 11.640, de 11 de janeiro de 2008)

CEUA
Comissão de Ética em Uso de Animais

Pró-Reitoria de Pesquisa, Pós-graduação e Inovação

COMISSÃO DE ÉTICA NO USO DE ANIMAIS - CEUA

Fone: (55) 3911-0200, E-mail:
ceua@unipampa.edu.br

TERMO DE CONSENTIMENTO

(conforme Resolução Normativa do CONCEA nº 22/2015)

Título do projeto:

Identificação e caracterização de *Leishmania* sp. em animais domésticos

Nome do pesquisador principal:

Irina Lubeck

Razão social e CIAEP instituição da CEUA que aprovou:

Universidade Federal do Pampa

Objetivos do estudo:

Geral

Verificar a ocorrência de *Leishmania* sp., em caninos, equinos e felinos de zona urbana e rural dos municípios de Barra do Quaraí e Uruguaiana – RS.

Específicos:

Verificar a presença de material genético de *Leishmania* sp. em amostras de sangue periférico e lesões de pele dos caninos, equinos e felinos, através da técnica de PCR;
Verificar a presença de material genético de *Leishmania* sp. em amostras de sangue periférico e lesões de pele dos caninos, equinos e felinos, através da técnica de qPCR;
Realizar a caracterização molecular em amostras positivas para verificar as espécies encontradas;
Avaliar os aspectos clínicos dos animais naturalmente infectados, visando um maior conhecimento acerca das possíveis manifestações clínicas;
Investigar a presença de anticorpos anti-*Leishmania* sp. nos caninos, equinos e felinos;
Investigar a presença de anticorpos anti-*Leishmania* sp. via ensaio de triagem imunocromatográfico Teste Rápido Dual Path Platform nos caninos;
Comparar as técnicas sorológicas e moleculares nas espécies animais estudadas.

Procedimentos a serem realizados com os animais:

Serão coletadas amostras de sangue via venopunção e coleta de suabes oculares para determinação da infecção por leishmania por métodos moleculares e sorológicos como também, será realizado exame clínico que consta de: aferição da frequência cardíaca, frequência respiratória, tempo de perfusão capilar, grau de hidratação, motilidade intestinal, inspeção de linfonodos, pulso digital e temperatura retal. Para tanto, será realizada somente uma coleta/visita. Animais que apresentem alterações clínicas como infartamento de linfonodos, se efetuada punção para realização de citologia. Em caso da ocorrência de lesões de pele, os animais serão encaminhados para o Hospital Veterinário Universitário da Unipampa (HUVet) para a realização de biópsia de pele.

Potenciais riscos para os animais:

Não são apresentados riscos aos animais

Cronograma:

Atividade	Set	Out	Nov	Dez	Jan	Fev	Mar	Abr	Mai
Coleta de felinos e caninos	X	X	X	X	X	X	X	X	X

Benefícios:

Os animais serão submetidos a exame clínico completo e avaliação por médico veterinário capacitado, permitindo a detecção de alterações clínicas e encaminhamento para investigações complementares. Adicionalmente, os animais são submetidos a testes para detecção de *Leishmania* ou resposta à mesma, fornecendo ao tutor informação sobre a situação de seu animal em relação a leishmaniose visceral e propiciando ao mesmo a tomada de ações com base no resultado do diagnóstico. As informações obtidas alertarão a população sobre a adoção de medidas preventivas, manejo com animais domésticos e manejo ambiental nas residências, evitando a disseminação do vetor e a ocorrência da enfermidade.

Esclarecimentos ao proprietário sobre a participação do animal neste projeto:

- Sua autorização para a inclusão do (s) seu (s) animal (is) nesse estudo é voluntária.
- Seu (s) animal (is) poderá(ão) ser retirado (s) do estudo, a qualquer momento, sem que isso cause qualquer prejuízo a ele (s).
- A confidencialidade dos seus dados pessoais será preservada.
- Os membros da CEUA ou as autoridades regulatórias poderão solicitar suas informações, e nesse caso, elas serão dirigidas especificamente para fins de inspeções regulares.

O Médico Veterinário responsável pelo (s) seu (s) anima (is) será o (a) Dr (a) Irina Lübeck, inscrita (a) no CRMV sob o n 8926. Além dele, a equipe do Pesquisador Principal Claudia Acosta Duarte também se responsabilizará pelo bem estar do (s) seu (s) animal (is) durante todo o estudo e ao final dele.

Quando for necessário, durante ou após o período do estudo, você poderá entrar em contato com o Pesquisador Principal ou com a sua equipe pelos contatos:

Tel. de emergência: (55) 999487512

Declaração de consentimento

Fui devidamente esclarecido (a) sobre todos os procedimentos deste estudo, seus riscos e benefícios ao (s) animal (is) pelo (s) qual (is) sou responsável. Fui também informado que posso retirar meu (s) animal (is) do estudo a qualquer momento. Ao assinar este Termo de Consentimento, declaro que autorizo a participação do (s) meu (s) animal (is) identificado (s), a seguir, neste projeto.

Local e data: Uruguaiana, Dezembro de 2017

Assinatura do Pesquisador: [signature]

Assinatura do Responsável: [signature]

MINISTÉRIO DA EDUCAÇÃO
FUNDAÇÃO UNIVERSIDADE FEDERAL DO PAMPA
(Lei nº 11.640, de 11 de janeiro de 2008)

CEUA
Comissão de Ética em Uso de Animais

Pró-Reitoria de Pesquisa, Pós-graduação e Inovação

COMISSÃO DE ÉTICA NO USO DE ANIMAIS - CEUA
Fone: (55) 3911-0200, E-mail:
ceua@unipampa.edu.br

TERMO DE CONSENTIMENTO
(conforme Resolução Normativa do CONCEA nº 22/2015)

Título do projeto:

Identificação e caracterização de *Leishmania* sp. em animais domésticos

Nome do pesquisador principal:

Irina Lubeck

Razão social e CIAEP instituição da CEUA que aprovou:

Universidade Federal do Pampa

Objetivos do estudo:

Geral

Verificar a ocorrência de *Leishmania* sp., em caninos, equinos e felinos de zona urbana e rural dos municípios de Barra do Quaraí e Uruguaiana – RS.

Específicos:

Verificar a presença de material genético de *Leishmania* sp. em amostras de sangue periférico e lesões de pele dos caninos, equinos e felinos, através da técnica de PCR;
Verificar a presença de material genético de *Leishmania* sp. em amostras de sangue periférico e lesões de pele dos caninos, equinos e felinos, através da técnica de qPCR;
Realizar a caracterização molecular em amostras positivas para verificar as espécies encontradas;
Avaliar os aspectos clínicos dos animais naturalmente infectados, visando um maior conhecimento acerca das possíveis manifestações clínicas;
Investigar a presença de anticorpos anti-*Leishmania* sp. nos caninos, equinos e felinos;
Investigar a presença de anticorpos anti-*Leishmania* sp. via ensaio de triagem imunocromatográfico Teste Rápido Dual Path Platform nos caninos;
Comparar as técnicas sorológicas e moleculares nas espécies animais estudadas.

Procedimentos a serem realizados com os animais:

Serão coletadas amostras de sangue via venopunção e coleta de suabes oculares para determinação da infecção por leishmania por métodos moleculares e sorológicos como também, será realizado exame clínico que consta de: aferição da frequência cardíaca, frequência respiratória, tempo de perfusão capilar, grau de hidratação, motilidade intestinal, inspeção de linfonodos, pulso digital e temperatura retal. Para tanto, será realizada somente uma coleta/visita. Animais que apresentem alterações clínicas como infartamento de linfonodos, se efetuada punção para realização de citologia. Em caso da ocorrência de lesões de pele, os animais serão encaminhados para o Hospital Veterinário Universitário da Unipampa (HUVet) para a realização de biópsia de pele.

Potenciais riscos para os animais:

Não são apresentados riscos aos animais

Cronograma:

Atividade	Set	Out	Nov	Dez	Jan	Fev	Mar	Abr	Mai
Coleta de felinos e caninos	X	X	X	X	X	X	X	X	X

Benefícios:

Os animais serão submetidos a exame clínico completo e avaliação por médico veterinário capacitado, permitindo a detecção de alterações clínicas e encaminhamento para investigações complementares. Adicionalmente, os animais são submetidos a testes para detecção de *Leishmania* ou resposta à mesma, fornecendo ao tutor informação sobre a situação de seu animal em relação a leishmaniose visceral e propiciando ao mesmo a tomada de ações com base no resultado do diagnóstico. As informações obtidas alertarão a população sobre a adoção de medidas preventivas, manejo com animais domésticos e manejo ambiental nas residências, evitando a disseminação do vetor e a ocorrência da enfermidade.

Esclarecimentos ao proprietário sobre a participação do animal neste projeto:

- Sua autorização para a inclusão do (s) seu (s) animal (is) nesse estudo é voluntária.
- Seu (s) animal (is) poderá(ão) ser retirado (s) do estudo, a qualquer momento, sem que isso cause qualquer prejuízo a ele (s).
- A confidencialidade dos seus dados pessoais será preservada.
- Os membros da CEUA ou as autoridades regulatórias poderão solicitar suas informações, e nesse caso, elas serão dirigidas especificamente para fins de inspeções regulares.

O Médico Veterinário responsável pelo (s) seu (s) anima (is) será o (a) Dr (a) Irina Lübeck, inscrita (a) no CRMV sob o n 8926. Além dele, a equipe do Pesquisador Principal Claudia Acosta Duarte também se responsabilizará pelo bem estar do (s) seu (s) animal (is) durante todo o estudo e ao final dele.

Quando for necessário, durante ou após o período do estudo, você poderá entrar em contato com o Pesquisador Principal ou com a sua equipe pelos contatos:

Tel. de emergência: (55) 999487512

Declaração de consentimento

Fui devidamente esclarecido (a) sobre todos os procedimentos deste estudo, seus riscos e benefícios ao (s) animal (is) pelo (s) qual (is) sou responsável. Fui também informado que posso retirar meu (s) animal (is) do estudo a qualquer momento. Ao assinar este Termo de Consentimento, declaro que autorizo a participação do (s) meu (s) animal (is) identificado (s), a seguir, neste projeto.

Local e data: Uruguaiana, 01 de novembro de 2017.

Assinatura do Pesquisador: [signature]

Assinatura do Responsável: [signature]

MINISTÉRIO DA EDUCAÇÃO
FUNDAÇÃO UNIVERSIDADE FEDERAL DO PAMPA
(Lei nº 11.640, de 11 de janeiro de 2008)

Pró-Reitoria de Pesquisa, Pós-graduação e Inovação
COMISSÃO DE ÉTICA NO USO DE ANIMAIS - CEUA
Fone: (55) 3911-0200, E-mail:
ceua@unipampa.edu.br

TERMO DE CONSENTIMENTO

(conforme Resolução Normativa do CONCEA nº 22/2015)

Título do projeto:

Identificação e caracterização de *Leishmania* sp. em animais domésticos

Nome do pesquisador principal:

Irina Lubeck

Razão social e CIAEP instituição da CEUA que aprovou:

Universidade Federal do Pampa

Objetivos do estudo:

Geral

Verificar a ocorrência de *Leishmania* sp., em caninos, equinos e felinos de zona urbana e rural dos municípios de Barra do Quaraí e Uruguaiana – RS.

Específicos:

Verificar a presença de material genético de *Leishmania* sp. em amostras de sangue periférico e lesões de pele dos caninos, equinos e felinos, através da técnica de PCR;
Verificar a presença de material genético de *Leishmania* sp. em amostras de sangue periférico e lesões de pele dos caninos, equinos e felinos, através da técnica de qPCR;
Realizar a caracterização molecular em amostras positivas para verificar as espécies encontradas;
Avaliar os aspectos clínicos dos animais naturalmente infectados, visando um maior conhecimento acerca das possíveis manifestações clínicas;
Investigar a presença de anticorpos anti-*Leishmania* sp. nos caninos, equinos e felinos;
Investigar a presença de anticorpos anti-*Leishmania* sp. via ensaio de triagem imunocromatográfico Teste Rápido Dual Path Platform nos caninos;
Comparar as técnicas sorológicas e moleculares nas espécies animais estudadas.

Procedimentos a serem realizados com os animais:

Serão coletadas amostras de sangue via venopunção e coleta de suabes oculares para determinação da infecção por leishmania por métodos moleculares e sorológicos como também, será realizado exame clínico que consta de: aferição da frequência cardíaca, frequência respiratória, tempo de perfusão capilar, grau de hidratação, motilidade intestinal, inspeção de linfonodos, pulso digital e temperatura retal. Para tanto, será realizada somente uma coleta/visita. Animais que apresentem alterações clínicas como infartamento de linfonodos, se efetuada punção para realização de citologia. Em caso da ocorrência de lesões de pele, os animais serão encaminhados para o Hospital Veterinário Universitário da Unipampa (HUVet) para a realização de biópsia de pele.

(nº de visitas, o que será realizado e quando, descrição do que será feito com os animais etc.)

Potenciais riscos para os animais:

Não são apresentados riscos aos animais

Cronograma:

Atividade	Set	Out	Nov	Dez	Jan	Fev	Mar	Abr	Mai

Coleta de felinos e caninos	X	X	X	X	X	X	X	X	X

Benefícios:

Os animais serão submetidos a exame clínico completo e avaliação por médico veterinário capacitado, permitindo a detecção de alterações clínicas e encaminhamento para investigações complementares. Adicionalmente, os animais são submetidos a testes para detecção de *Leishmania* ou resposta à mesma, fornecendo ao tutor informação sobre a situação de seu animal em relação a leishmaniose visceral e propiciando ao mesmo a tomada de ações com base no resultado do diagnóstico. As informações obtidas alertarão a população sobre a adoção de medidas preventivas, manejo com animais domésticos e manejo ambiental nas residências, evitando a disseminação do vetor e a ocorrência da enfermidade.

Descrever os benefícios do estudo para o animal e, se for o caso, para outros animais que poderão se beneficiar com os resultados do projeto.
Se houver algum benefício para a sociedade, o pesquisador também deve mencionar.

Esclarecimentos ao proprietário sobre a participação do animal neste projeto:

- Sua autorização para a inclusão do (s) seu (s) animal (is) nesse estudo é voluntária.
- Seu (s) animal (is) poderá(ão) ser retirado (s) do estudo, a qualquer momento, sem que isso cause qualquer prejuízo a ele (s).
- A confidencialidade dos seus dados pessoais será preservada.
- Os membros da CEUA ou as autoridades regulatórias poderão solicitar suas informações, e nesse caso, elas serão dirigidas especificamente para fins de inspeções regulares.

O Médico Veterinário responsável pelo (s) seu (s) anima (is) será o (a) Dr (a) Irina Lubeck, inscrita (a) no CRMV sob o n 8926. Além dele, a equipe do Pesquisador Principal Claudia Acosta Duarte também se responsabilizará pelo bem estar do (s) seu (s) animal (is) durante todo o estudo e ao final dele.
Quando for necessário, durante ou após o período do estudo, você poderá entrar em contato com o Pesquisador Principal ou com a sua equipe pelos contatos:

Tel. de emergência: (55) 999487512

Declaração de consentimento

Fui devidamente esclarecido (a) sobre todos os procedimentos deste estudo, seus riscos e benefícios ao (s) animal (is) pelo (s) qual (is) sou responsável. Fui também informado que posso retirar meu (s) animal (is) do estudo a qualquer momento. Ao assinar este Termo de Consentimento, declaro que autorizo a participação do (s) meu (s) animal (is) identificado (s), a seguir, neste projeto.

Este documento será assinado em duas vias, sendo que uma via ficará comigo e outra com o pesquisador.

URUGUAIANA, 27/01/2021

Assinatura do Pesquisador: ______________________

Assinatura do Responsável: Louana R. Feratto ______________________

MINISTÉRIO DA EDUCAÇÃO
FUNDAÇÃO UNIVERSIDADE FEDERAL DO PAMPA
(Lei nº 11.640, de 11 de janeiro de 2008)

CEUA

Pró-Reitoria de Pesquisa, Pós-graduação e Inovação

COMISSÃO DE ÉTICA NO USO DE ANIMAIS - CEUA
Fone: (55) 3911-0200, E-mail:
ceua@unipampa.edu.br

TERMO DE CONSENTIMENTO
(conforme Resolução Normativa do CONCEA nº 22/2015)

Título do projeto:

Identificação e caracterização de *Leishmania* sp. em animais domésticos

Nome do pesquisador principal:

Irina Lubeck

Razão social e CIAEP instituição da CEUA que aprovou:

Universidade Federal do Pampa

Objetivos do estudo:

Geral

Verificar a ocorrência de *Leishmania* sp., em caninos, equinos e felinos de zona urbana e rural dos municípios de Barra do Quaraí e Uruguaiana – RS.

Específicos:

Verificar a presença de material genético de *Leishmania* sp. em amostras de sangue periférico e lesões de pele dos caninos, equinos e felinos, através da técnica de PCR;
Verificar a presença de material genético de *Leishmania* sp. em amostras de sangue periférico e lesões de pele dos caninos, equinos e felinos, através da técnica de qPCR;
Realizar a caracterização molecular em amostras positivas para verificar as espécies encontradas;
Avaliar os aspectos clínicos dos animais naturalmente infectados, visando um maior conhecimento acerca das possíveis manifestações clínicas;
Investigar a presença de anticorpos anti-*Leishmania* sp. nos caninos, equinos e felinos;
Investigar a presença de anticorpos anti-*Leishmania* sp. via ensaio de triagem imunocromatográfico Teste Rápido Dual Path Platform nos caninos;
Comparar as técnicas sorológicas e moleculares nas espécies animais estudadas.

Procedimentos a serem realizados com os animais:

Serão coletadas amostras de sangue via venopunção e coleta de suabes oculares para determinação da infecção por leishmania por métodos moleculares e sorológicos como também, será realizado exame clínico que consta de: aferição da frequência cardíaca, frequência respiratória, tempo de perfusão capilar, grau de hidratação, motilidade intestinal, inspeção de linfonodos, pulso digital e temperatura retal. Para tanto, será realizada somente uma coleta/visita. Animais que apresentem alterações clínicas como infartamento de linfonodos, se efetuada punção para realização de citologia. Em caso da ocorrência de lesões de pele, os animais serão encaminhados para o Hospital Veterinário Universitário da Unipampa (HUVet) para a realização de biópsia de pele.

Potenciais riscos para os animais:

Não são apresentados riscos aos animais

Cronograma:

Atividade	Set	Out	Nov	Dez	Jan	Fev	Mar	Abr	Mai
Coleta de felinos e caninos	X	X	X	X	X	X	X	X	X

Benefícios:

Os animais serão submetidos a exame clínico completo e avaliação por médico veterinário capacitado, permitindo a detecção de alterações clínicas e encaminhamento para investigações complementares. Adicionalmente, os animais são submetidos a testes para detecção de *Leishmania* ou resposta à mesma, fornecendo ao tutor informação sobre a situação de seu animal em relação a leishmaniose visceral e propiciando ao mesmo a tomada de ações com base no resultado do diagnóstico. As informações obtidas alertarão a população sobre a adoção de medidas preventivas, manejo com animais domésticos e manejo ambiental nas residências, evitando a disseminação do vetor e a ocorrência da enfermidade.

Esclarecimentos ao proprietário sobre a participação do animal neste projeto:

- Sua autorização para a inclusão do (s) seu (s) animal (is) nesse estudo é voluntária.
- Seu (s) animal (is) poderá(ão) ser retirado (s) do estudo, a qualquer momento, sem que isso cause qualquer prejuízo a ele (s).
- A confidencialidade dos seus dados pessoais será preservada.
- Os membros da CEUA ou as autoridades regulatórias poderão solicitar suas informações, e nesse caso, elas serão dirigidas especificamente para fins de inspeções regulares.

O Médico Veterinário responsável pelo (s) seu (s) anima (is) será o (a) Dr (a) Irina Lübeck, inscrita (a) no CRMV sob o n 8926. Além dele, a equipe do Pesquisador Principal Claudia Acosta Duarte também se responsabilizará pelo bem estar do (s) seu (s) animal (is) durante todo o estudo e ao final dele.
Quando for necessário, durante ou após o período do estudo, você poderá entrar em contato com o Pesquisador Principal ou com a sua equipe pelos contatos:

Tel. de emergência: (55) 999487512

Declaração de consentimento

Fui devidamente esclarecido (a) sobre todos os procedimentos deste estudo, seus riscos e benefícios ao (s) animal (is) pelo (s) qual (is) sou responsável. Fui também informado que posso retirar meu (s) animal (is) do estudo a qualquer momento. Ao assinar este Termo de Consentimento, declaro que autorizo a participação do (s) meu (s) animal (is) identificado (s), a seguir, neste projeto.

Local e data: URUGUAIANA, RS, DE JULHO DE 2017.

Assinatura do Pesquisador: [signature]

Assinatura do Responsável: [signature]

MINISTÉRIO DA EDUCAÇÃO
FUNDAÇÃO UNIVERSIDADE FEDERAL DO PAMPA
(Lei nº 11.640, de 11 de janeiro de 2008)

CEUA
Comissão de Ética em Uso de Animais

Pró-Reitoria de Pesquisa, Pós-graduação e Inovação

COMISSÃO DE ÉTICA NO USO DE ANIMAIS - CEUA
Fone: (55) 3911-0200, E-mail: ceua@unipampa.edu.br

TERMO DE CONSENTIMENTO
(conforme Resolução Normativa do CONCEA nº 22/2015)

Título do projeto:
Identificação e caracterização de *Leishmania* sp. em animais domésticos

Nome do pesquisador principal:
Irina Lubeck

Razão social e CIAEP instituição da CEUA que aprovou:
Universidade Federal do Pampa

Objetivos do estudo:
Geral
Verificar a ocorrência de *Leishmania* sp., em caninos, equinos e felinos de zona urbana e rural dos municípios de Barra do Quaraí e Uruguaiana – RS.
Específicos:
Verificar a presença de material genético de *Leishmania* sp. em amostras de sangue periférico e lesões de pele dos caninos, equinos e felinos, através da técnica de PCR;
Verificar a presença de material genético de *Leishmania* sp. em amostras de sangue periférico e lesões de pele dos caninos, equinos e felinos, através da técnica de qPCR;
Realizar a caracterização molecular em amostras positivas para verificar as espécies encontradas;
Avaliar os aspectos clínicos dos animais naturalmente infectados, visando um maior conhecimento acerca das possíveis manifestações clínicas;
Investigar a presença de anticorpos anti-*Leishmania* sp. nos caninos, equinos e felinos;
Investigar a presença de anticorpos anti-*Leishmania* sp. via ensaio de triagem imunocromatográfico Teste Rápido Dual Path Platform nos caninos;
Comparar as técnicas sorológicas e moleculares nas espécies animais estudadas.

Procedimentos a serem realizados com os animais:
Serão coletadas amostras de sangue via venopunção e coleta de suabes oculares para determinação da infecção por leishmania por métodos moleculares e sorológicos como também, será realizado exame clínico que consta de: aferição da frequência cardíaca, frequência respiratória, tempo de perfusão capilar, grau de hidratação, motilidade intestinal, inspeção de linfonodos, pulso digital e temperatura retal. Para tanto, será realizada somente uma coleta/visita. Animais que apresentem alterações clínicas como infartamento de linfonodos, se efetuada punção para realização de citologia. Em caso da ocorrência de lesões de pele, os animais serão encaminhados para o Hospital Veterinário Universitário da Unipampa (HUVet) para a realização de biópsia de pele.

Potenciais riscos para os animais:
Não são apresentados riscos aos animais

Cronograma:

Atividade	Set	Out	Nov	Dez	Jan	Fev	Mar	Abr	Mai
Coleta de felinos e caninos	X	X	X	X	X	X	X	X	X

Benefícios:

Os animais serão submetidos a exame clínico completo e avaliação por médico veterinário capacitado, permitindo a detecção de alterações clínicas e encaminhamento para investigações complementares. Adicionalmente, os animais são submetidos a testes para detecção de *Leishmania* ou resposta à mesma, fornecendo ao tutor informação sobre a situação de seu animal em relação a leishmaniose visceral e propiciando ao mesmo a tomada de ações com base no resultado do diagnóstico. As informações obtidas alertarão a população sobre a adoção de medidas preventivas, manejo com animais domésticos e manejo ambiental nas residências, evitando a disseminação do vetor e a ocorrência da enfermidade.

Esclarecimentos ao proprietário sobre a participação do animal neste projeto:

– Sua autorização para a inclusão do (s) seu (s) animal (is) nesse estudo é voluntária.
– Seu (s) animal (is) poderá(ão) ser retirado (s) do estudo, a qualquer momento, sem que isso cause qualquer prejuízo a ele (s).
– A confidencialidade dos seus dados pessoais será preservada.
– Os membros da CEUA ou as autoridades regulatórias poderão solicitar suas informações, e nesse caso, elas serão dirigidas especificamente para fins de inspeções regulares.

O Médico Veterinário responsável pelo (s) seu (s) anima (is) será o (a) Dr (a) Irina Lübeck, inscrita (a) no CRMV sob o n 8926. Além dele, a equipe do Pesquisador Principal Claudia Acosta Duarte também se responsabilizará pelo bem estar do (s) seu (s) animal (is) durante todo o estudo e ao final dele.
Quando for necessário, durante ou após o período do estudo, você poderá entrar em contato com o Pesquisador Principal ou com a sua equipe pelos contatos:

Tel. de emergência: (55) 999487512

Declaração de consentimento

Fui devidamente esclarecido (a) sobre todos os procedimentos deste estudo, seus riscos e benefícios ao (s) animal (is) pelo (s) qual (is) sou responsável. Fui também informado que posso retirar meu (s) animal (is) do estudo a qualquer momento. Ao assinar este Termo de Consentimento, declaro que autorizo a participação do (s) meu (s) animal (is) identificado (s), a seguir, neste projeto.

Local e data: Uruguaiana, dezembro 2017

Assinatura do Pesquisador: [signature]

Assinatura do Responsável: [signature]

MINISTÉRIO DA EDUCAÇÃO
FUNDAÇÃO UNIVERSIDADE FEDERAL DO PAMPA
(Lei nº 11.640, de 11 de janeiro de 2008)

CEUA
Comissão de Ética em Uso de Animais

Pró-Reitoria de Pesquisa, Pós-graduação e Inovação

COMISSÃO DE ÉTICA NO USO DE ANIMAIS - CEUA
Fone: (55) 3911-0200, E-mail:
ceua@unipampa.edu.br

TERMO DE CONSENTIMENTO
(conforme Resolução Normativa do CONCEA nº 22/2015)

Título do projeto:
Identificação e caracterização de *Leishmania* sp. em animais domésticos

Nome do pesquisador principal:
Irina Lubeck

Razão social e CIAEP instituição da CEUA que aprovou:
Universidade Federal do Pampa

Objetivos do estudo:

Geral
Verificar a ocorrência de *Leishmania* sp., em caninos, equinos e felinos de zona urbana e rural dos municípios de Barra do Quaraí e Uruguaiana – RS.

Específicos:
Verificar a presença de material genético de *Leishmania* sp. em amostras de sangue periférico e lesões de pele dos caninos, equinos e felinos, através da técnica de PCR;
Verificar a presença de material genético de *Leishmania* sp. em amostras de sangue periférico e lesões de pele dos caninos, equinos e felinos, através da técnica de qPCR;
Realizar a caracterização molecular em amostras positivas para verificar as espécies encontradas;
Avaliar os aspectos clínicos dos animais naturalmente infectados, visando um maior conhecimento acerca das possíveis manifestações clínicas;
Investigar a presença de anticorpos anti-*Leishmania* sp. nos caninos, equinos e felinos;
Investigar a presença de anticorpos anti-*Leishmania* sp. via ensaio de triagem imunocromatográfico Teste Rápido Dual Path Platform nos caninos;
Comparar as técnicas sorológicas e moleculares nas espécies animais estudadas.

Procedimentos a serem realizados com os animais:
Serão coletadas amostras de sangue via venopunção e coleta de suabes oculares para determinação da infecção por leishmania por métodos moleculares e sorológicos como também, será realizado exame clínico que consta de: aferição da frequência cardíaca, frequência respiratória, tempo de perfusão capilar, grau de hidratação, motilidade intestinal, inspeção de linfonodos, pulso digital e temperatura retal. Para tanto, será realizada somente uma coleta/visita. Animais que apresentem alterações clínicas como infartamento de linfonodos, se efetuada punção para realização de citologia. Em caso da ocorrência de lesões de pele, os animais serão encaminhados para o Hospital Veterinário Universitário da Unipampa (HUVet) para a realização de biópsia de pele.

Potenciais riscos para os animais:
Não são apresentados riscos aos animais

Cronograma:

Atividade	Set	Out	Nov	Dez	Jan	Fev	Mar	Abr	Mai
Coleta de felinos e caninos	X	X	X	X	X	X	X	X	X

Benefícios:

Os animais serão submetidos a exame clínico completo e avaliação por médico veterinário capacitado, permitindo a detecção de alterações clínicas e encaminhamento para investigações complementares. Adicionalmente, os animais são submetidos a testes para detecção de *Leishmania* ou resposta à mesma, fornecendo ao tutor informação sobre a situação de seu animal em relação a leishmaniose visceral e propiciando ao mesmo a tomada de ações com base no resultado do diagnóstico. As informações obtidas alertarão a população sobre a adoção de medidas preventivas, manejo com animais domésticos e manejo ambiental nas residências, evitando a disseminação do vetor e a ocorrência da enfermidade.

Esclarecimentos ao proprietário sobre a participação do animal neste projeto:

- Sua autorização para a inclusão do (s) seu (s) animal (is) nesse estudo é voluntária.
- Seu (s) animal (is) poderá(ão) ser retirado (s) do estudo, a qualquer momento, sem que isso cause qualquer prejuízo a ele (s).
- A confidencialidade dos seus dados pessoais será preservada.
- Os membros da CEUA ou as autoridades regulatórias poderão solicitar suas informações, e nesse caso, elas serão dirigidas especificamente para fins de inspeções regulares.

O Médico Veterinário responsável pelo (s) seu (s) anima (is) será o (a) Dr (a) Irina Lübeck, inscrita (a) no CRMV sob o n 8926. Além dele, a equipe do Pesquisador Principal Claudia Acosta Duarte também se responsabilizará pelo bem estar do (s) seu (s) animal (is) durante todo o estudo e ao final dele.
Quando for necessário, durante ou após o período do estudo, você poderá entrar em contato com o Pesquisador Principal ou com a sua equipe pelos contatos:

Tel. de emergência: (55) 999487512

Declaração de consentimento

Fui devidamente esclarecido (a) sobre todos os procedimentos deste estudo, seus riscos e benefícios ao (s) animal (is) pelo (s) qual (is) sou responsável. Fui também informado que posso retirar meu (s) animal (is) do estudo a qualquer momento. Ao assinar este Termo de Consentimento, declaro que autorizo a participação do (s) meu (s) animal (is) identificado (s), a seguir, neste projeto.

Local e data: Uruguaiana, Julho de 2017

Assinatura do Pesquisador: [signature]

Assinatura do Responsável: [signature]
