# Supplementary figures and images for: Risk and protective factors of Leishmaniasis in the rural area of the western border region of Rio Grande do Sul, Brazil
Source: BMC Vet Res. 2021 Oct 14;17:330. doi: 10.1186/s12917-021-03021-6 (PMC8515718; doi:10.1186/s12917-021-03021-6)

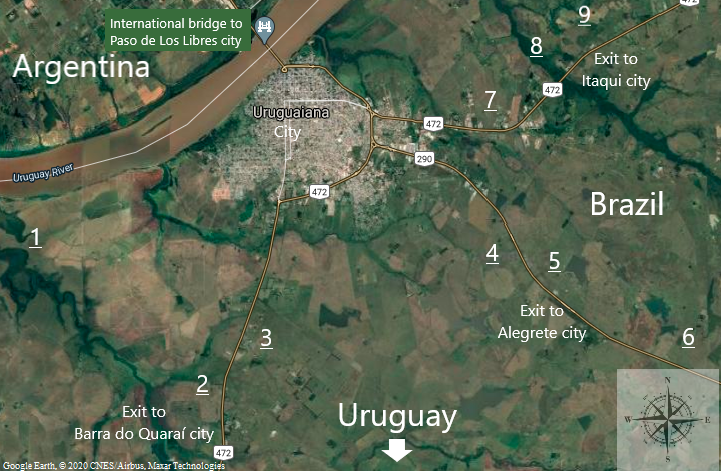

Supplement: Supplementary file 5 — Additional file 5. Fig. 1. Demonstrative map of the distribution of rural properties on the Western Frontier of Rio Grande do Sul (numbered from 1 to 9) where data and biological material collections were carried out. Maps data: Google Earth, © 2020 CNES/Airbus, Maxar Technologies. [file 12917_2021_3021_MOESM5_ESM.png]

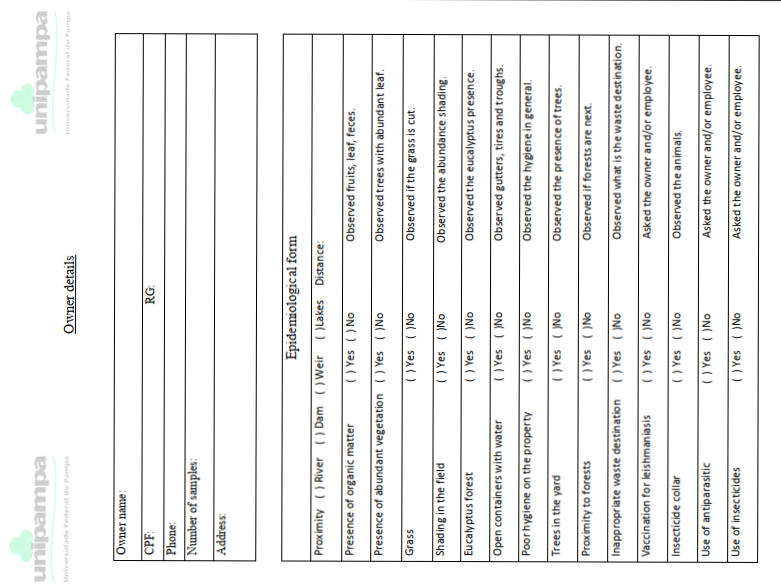

Supplement: Supplementary file 6 — Additional file 6. Fig. 2. Epidemiological form used to register the environmental variables. [file 12917_2021_3021_MOESM6_ESM.png]
