# Supplementary material for: Risk and protective factors of Leishmaniasis in the rural area of the western border region of Rio Grande do Sul, Brazil
Source: BMC Vet Res. 2021 Oct 14;17:330. doi: 10.1186/s12917-021-03021-6 (PMC8515718; doi:10.1186/s12917-021-03021-6)
Supplement: Supplementary file 7 — Additional file 7. [file 12917_2021_3021_MOESM7_ESM.pdf]

GOVERNO DO ESTADO
RIO GRANDE DO SUL
SECRETARIA DA SAÚDE

NOTA TÉCNICA CONJUNTA Nº 01/2014 – CEVS – IPB-LACEN – SES/RS

LEISHMANIOSE VISCERAL NO ESTADO DO RIO GRANDE DO SUL

A Leishmaniose Visceral (LV) é uma doença causada por protozoários do gênero *Leishmania* e nas Américas, pela espécie *Leishmania chagasi*. A forma de transmissão é por meio da picada de fêmeas de insetos flebotomíneos infectados, principalmente, por *Lutzomyia longipalpis*.

Inicialmente caracterizada como doença eminentemente rural, mais recentemente vem se expandindo para áreas urbanas de médio e grande porte e se tornou crescente problema de saúde pública no país, sendo uma endemia em franca expansão geográfica.

Até o ano de 2007, o Rio Grande do Sul era considerado área indene para Leishmaniose Visceral. No entanto, em outubro de 2008, um cão residente no município de São Borja foi diagnosticado com Leishmaniose Visceral. Nos meses de novembro e dezembro seguintes, capturas de flebotomíneos foram efetuadas na cidade para corroborar a autoctonia do caso canino e comprovaram a presença do vetor *Lutzomyia longipalpis.* Já em janeiro de 2009, foi confirmado o primeiro caso humano autóctone do município.

O programa de Vigilância da Leishmaniose Visceral está dividido em:

1 - Leishmaniose Visceral Humana (LVH)

Situação epidemiológica

A Leishmaniose Visceral Humana (LVH) é uma doença endêmica em 88 países da região tropical e subtropical. É uma antropozoonose considerada inicialmente de transmissão silvestre, com características de ambientes rurais, mas, atualmente está ocorrendo nas áreas periurbanas e urbanas.

É um crescente problema de saúde pública no Brasil e encontra-se em franca expansão geográfica, estando distribuída em 21 unidades federadas, atingindo as cinco regiões brasileiras.

Segundo dados do Ministério da Saúde, no período entre 2003 e 2012, foram registrados 35.248 casos da LVH no país. Em 2012, a região Nordeste representou

43,1% dos casos, seguida pelas regiões Norte (19,6%), Sudeste (16,7%), Centro-Oeste (11,6%) e o Sul com um caso que representa menor que um por cento (0,03%). A doença é mais freqüente no sexo masculino e em crianças menores de 10 anos.

Em janeiro de 2009, a SES/RS notificou o primeiro caso humano autóctone confirmado da LVH no município de São Borja, em paciente adulto, do sexo masculino com início dos sintomas no final do ano de 2008 e com evolução para cura após tratamento.

No Rio Grande do Sul, no período de janeiro de 2009 a dezembro de 2012, foram confirmados 12 casos da LVH, sendo sete casos autóctones do município de São Borja, dois de Itaqui e um de Uruguaiana e outros dois casos importados de outros estados do país.

Técnicas diagnósticas

O diagnóstico laboratorial imunológico, de rotina, mais utilizado no Brasil é a Reação de Imunofluorescência Indireta (RIFI), anticorpo pesquisado: IgG. É considerado positivo o resultado da RIFI nas diluições a partir de 1:80. Títulos variáveis podem persistir positivos mesmo após o tratamento. Nos títulos iguais a 1:40, é recomendada a solicitação de nova amostra em 30 dias. Na presença de dados clínicos e laboratoriais, um teste sorológico reagente reforça o diagnóstico. No entanto, um teste reagente não autoriza o tratamento, na *ausência de manifestações clínicas sugestivas da LVH.*

Entre outras técnicas laboratoriais, o diagnóstico parasitológico, considerado como de certeza, é feito pelo encontro de formas amastigotas do parasito em material biológico obtido preferencialmente da medula óssea (por ser um procedimento mais seguro), aspiração de linfonodos ou biópsia hepática. O método do PCR (amplificação do DNA do parasito) apresenta alta especificidade e a amostra pode ser o sangue total. Estas três técnicas são oferecidas na rede pública de saúde.

Outra alternativa para o diagnóstico é o teste imunocromatográfico (teste rápido), o qual detecta anticorpos de LVH. Tem como vantagem à praticidade da execução e interpretação dos resultados, determinando na hora se o paciente está ou não com Leishmaniose. Além disso, apresenta alta especificidade. Esta metodologia está disponível nos municípios para agilizar o diagnóstico. Em caso positivo é feita a contraprova com soro (RIFI).

As amostras enviadas ao IPB-LACEN/RS devem estar acompanhadas da ficha do GAL e requisição médica.

Tratamento

As drogas atualmente preconizadas para o tratamento da leishmaniose visceral são: o antimoniato de N-metil glucamina, a anfotericina B (desoxicolato de anfotericina B e a anfotericina B lipossomal) e o isotionato de pentamidina. A escolha da droga baseia-se na sua toxicidade e eficácia. Estes medicamentos são considerados estratégicos e fornecidos pelo Ministério da Saúde. O antimoniato de N-metil glucamina e o desoxicolato de anfotericina B são disponibilizados mediante receituário médico pela Secretaria Estadual da Saúde/CEVS, enquanto a anfotericina B lipossomal tem formulário próprio para solicitação ao Ministério da Saúde/Secretaria Estadual da Saúde.

Mais informações sobre o tratamento no site:
http://portalsaude.saude.gov.br/index.php/o-ministerio/principal/leia-mais-o-ministerio/727-secretaria-svs/vigilancia-de-a-a-z/leishmaniose-visceral-lv/l1-leishmaniose-visceral-lv/14190-orientacoes-para-uso-racional-do-medicamento-anfotericina-b-lipossomal

2 - Leishmaniose Visceral Canina (LVC)

A transmissão da LVC, no Rio Grande do Sul, está ocorrendo nos municípios de Uruguaiana, Itaqui, São Borja, Porto Alegre e Santa Cruz do Sul. A partir do surgimento de novas áreas de transmissão da LVC, a Vigilância Estadual do Agravo esclarece:

- O cão doméstico é o principal reservatório do parasito no meio urbano e a principal fonte de alimento para o vetor. A doença em cães geralmente precede a ocorrência de casos humanos, por isso a necessidade de controle da LVC, a fim de evitar casos humanos e de outros cães.

- Animais suspeitos da LVC têm o sangue testado pelo Laboratório Central do Estado (LACEN), utilizando o teste rápido imunocromatográfico (triagem) e técnica de ELISA (confirmatório) (Nota Técnica do Ministério da Saúde Nº1/11). Quando os dois testes são positivos, o cão é considerado portador da doença (sintomático ou assintomático), sendo que a única medida recomendada é a eutanásia, segundo as normas Programa de Vigilância e Controle da Leishmaniose Visceral (PVCLV) do Ministério da Saúde.

- A Portaria interministerial nº 1.426, de 11 de julho de 2008 – "Proíbe o tratamento da leishmaniose visceral canina com produtos de uso humano ou não registrados no Ministério da Agricultura, Pecuária e Abastecimento".

- O Ministério da Saúde e Secretaria Estadual da Saúde/RS não reconhecem a vacina e/ou a coleira impregnada com inseticida como estratégias de controle da transmissão da LVC.

3 - Vigilância Entomológica (VE)

O vetor *Lutzomyia longipalpis* foi capturado nos municípios de Barra do Quaraí, Garruchos, Porto Xavier, Pirapó, Uruguaiana, Itaqui e São Borja, sendo que, nos três últimos, há transmissão do parasito pelo vetor. Santa Cruz do Sul e Porto Alegre são municípios com transmissão da LVC, mas, até o momento, não foi encontrado *Lutzomyia longipalpis.*

O controle químico dos vetores somente é utilizado em torno da área de infecção de casos humanos, não sendo recomendado em áreas silvestres.

Medidas de Proteção Individual

- As recomendações que podem diminuir o contato do homem com o vetor são: uso de repelente, evitar áreas onde há transmissão da doença, não se expor nos horários de atividade do vetor (crepúsculo e noite) em ambientes infestados e utilização de tela mosquiteira com malha fina.

Medidas de Saneamento Ambiental

– Consistem em manter, de forma contínua planificada, o ambiente em condições limpas e desfavoráveis para o estabelecimento de criadouros de formas imaturas e adultas do vetor, por meio da limpeza de quintais, terrenos e praças públicas, eliminação da fonte de umidade e dos resíduos sólidos orgânicos, retirada de entulhos e materiais inservíveis e sua destinação adequada.

Porto Alegre, 18 de agosto de 2014
